# Supplementary figures and images for: Microvasculopathy and soft tissue calcification in mice are governed by fetuin-A, magnesium and pyrophosphate
Source: PLoS One. 2020 Feb 19;15(2):e0228938. doi: 10.1371/journal.pone.0228938 (PMC7029863; doi:10.1371/journal.pone.0228938)

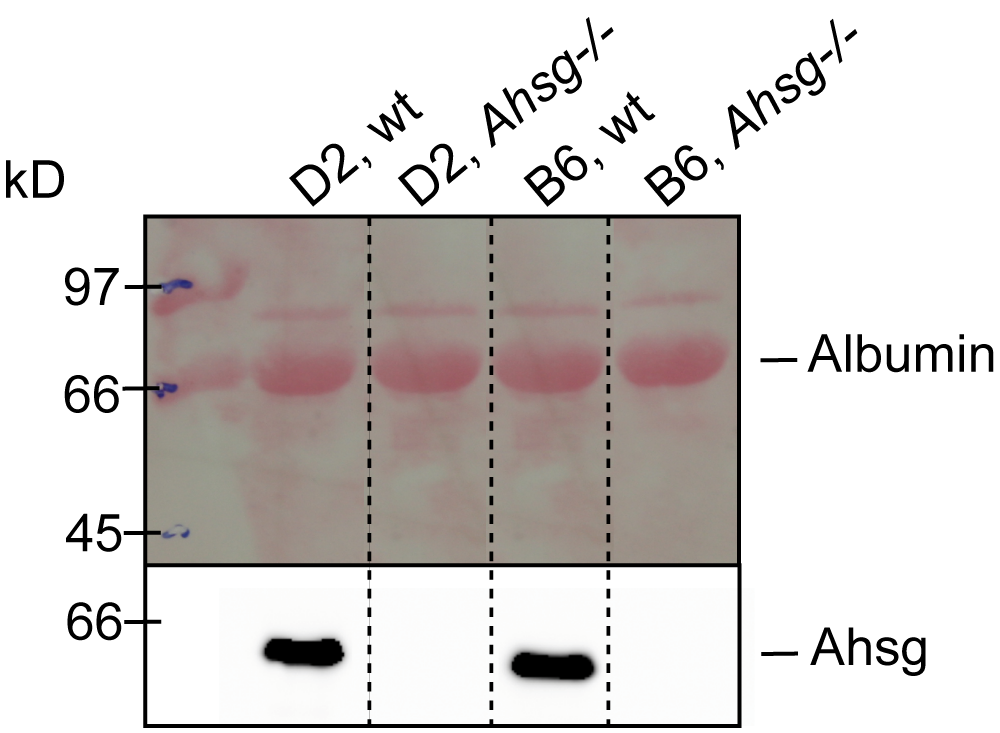

Supplement: S1 Fig — (JPG) [file pone.0228938.s001.jpg]

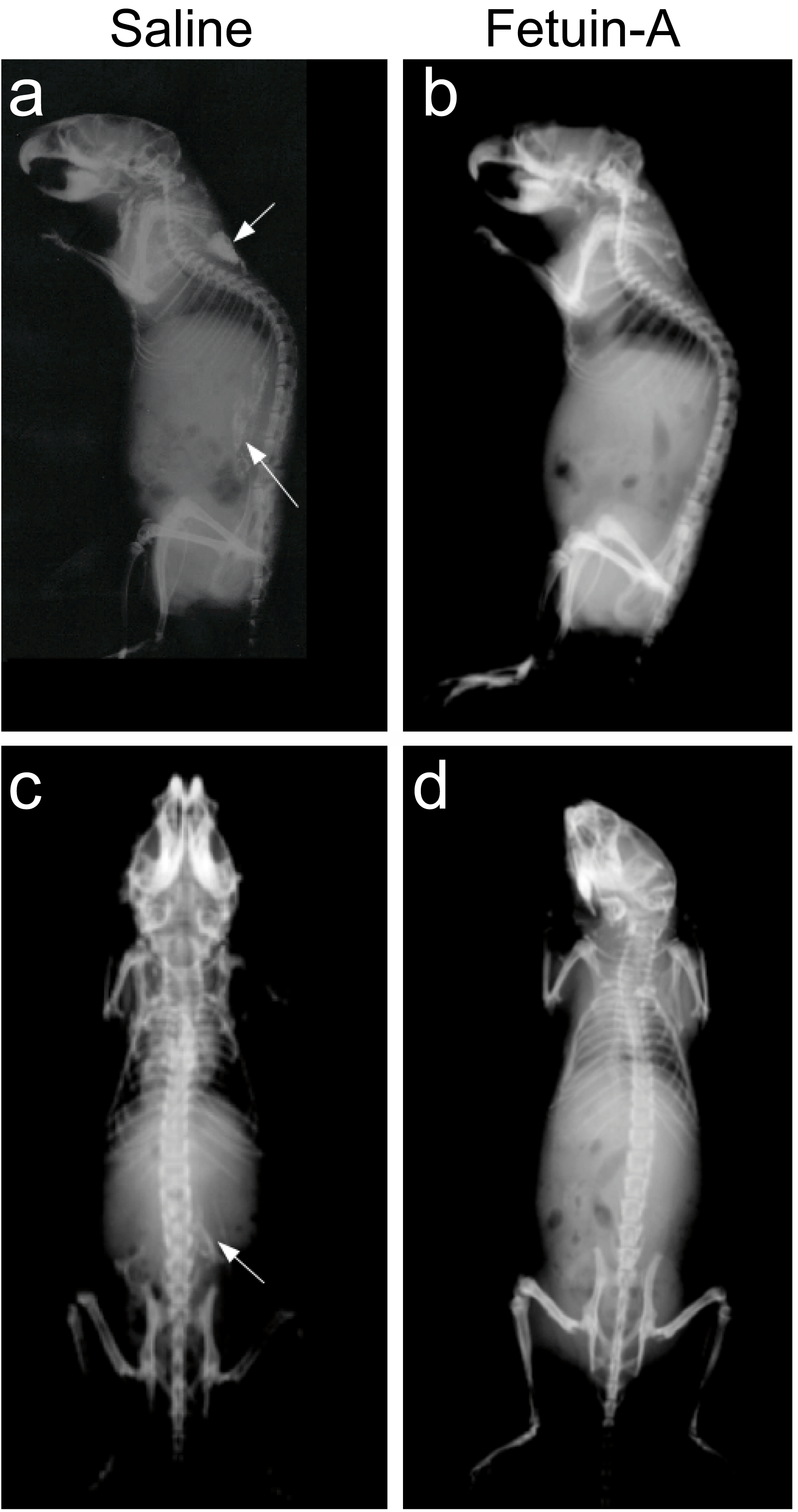

Supplement: S2 Fig — a-d, Three-week-old mice were injected i.p. five times a week for three weeks, with saline (a,c) or with 0.24 g/kg bodyweight fetuin-A (b,d). Lateral and dorsal radiographs show brown adipose tissue and renal fat calcification (arrows) in saline treated but not in fetuin-A treated mice. (JPG) [file pone.0228938.s002.jpg]

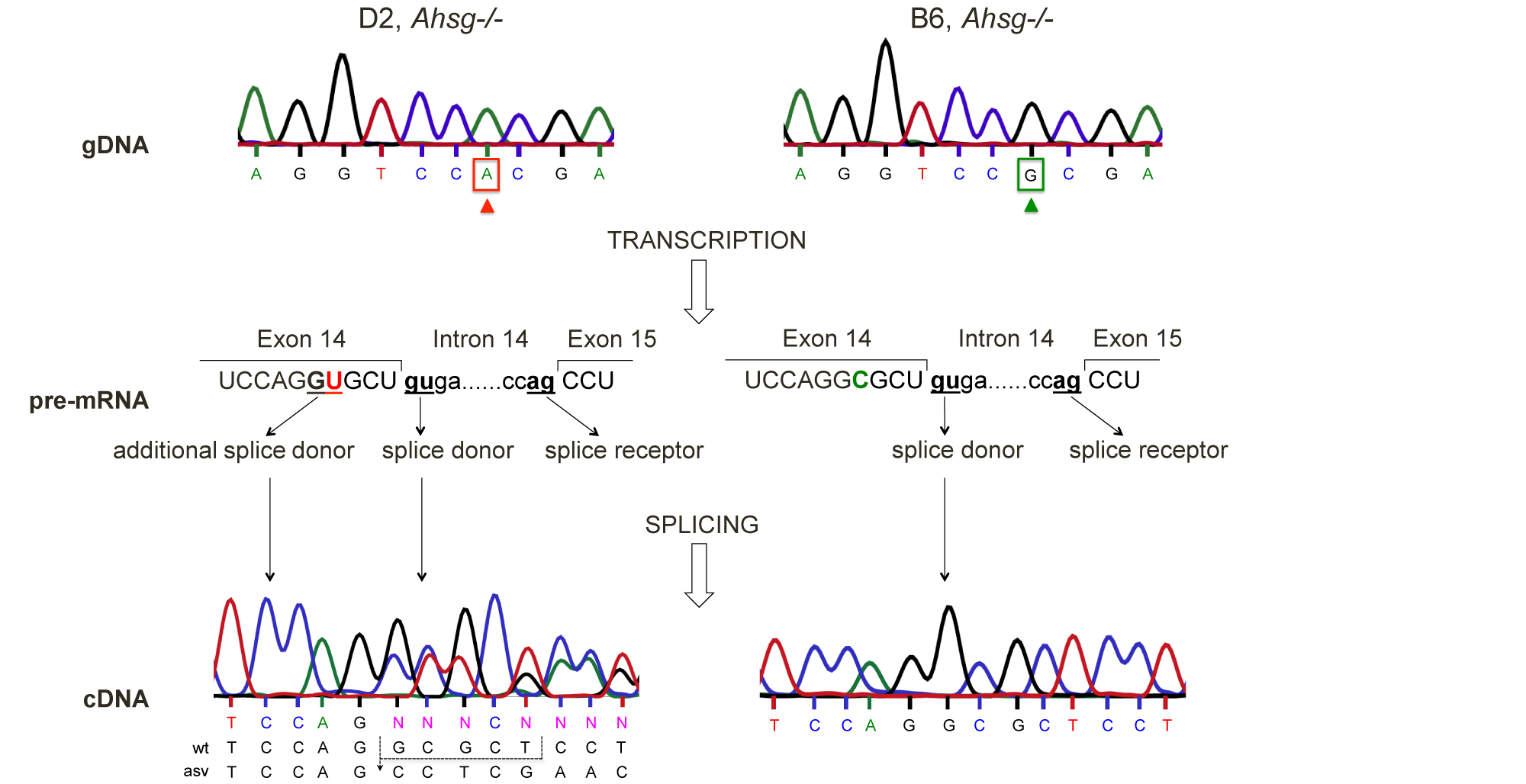

Supplement: S3 Fig — B6 mice have unambiguous splice donor and acceptor sites in their genomic DNA (gDNA) resulting in a single pre-mRNA and a single mRNA transcript. In contrast, D2 mice carry a G>A mutation in their gDNA, creating an additional splice donor site five base pairs upstream of the wildtype splice donor site. This creates an alternative five base pairs shortened splice variant of the mRNA transcript, which is not translated. Thus, the hypomorphic SNP rs32756904 results in reduced expression of functional Abcc6 protein (Fig 6D) and reduced extracellular pyrophosphate levels (Fig 1I). (JPG) [file pone.0228938.s003.jpg]
