# Supplementary material for: Microvasculopathy and soft tissue calcification in mice are governed by fetuin-A, magnesium and pyrophosphate
Source: PLoS One. 2020 Feb 19;15(2):e0228938. doi: 10.1371/journal.pone.0228938 (PMC7029863; doi:10.1371/journal.pone.0228938)
Supplement: S1 Table — (DOCX) [file pone.0228938.s004.docx]

**S1 Table. Primer sequences used for quantitative real-time PCR.** Primers were designed using NCBI primer-BLAST or taken from Primer Bank. We ensured that all primers were spanning exon-exon junctions and primed the coding region of the gene.

| Primer | Fragment size [bp] | Sequence (5` → 3`) |
| --- | --- | --- |
| Abcc6_for | 104 | GCATCGTTCAGGCTCGAGTG |
| Abcc6_rev |  | CGAGGAGCGCCTGGAGTTAC |
| Trpm6_for | 159 | TCTGCCACAATTTAGTCAGGTG |
| Trpm6_rev |  | TGGTGCCGAAGGTATCTGTAG |
| Trpm7_for | 128 | AGGATGTCAGATTTGTCAGCAAC |
| Trpm7_rev |  | CCTGGTTAAAGTGTTCACCCAA |
| Gapdh_for | 70 | AGATGGTGATGGGCTTCCC |
| Gapdh_rev |  | GGCAAATTCAACGGCACAGT |
